# Impact of vacuum assisted wound therapy on wound complications in STS surgery- A 3-year retrospective single-centre analysis

**DOI:** 10.1007/s00423-025-03865-8

**Published:** 2025-09-25

**Authors:** Simone Schewe, Adrian Sagebiel, Jonas Wakker, Nina Voss, Ruba Al Shonikat, Matthias Priemel, Alonja Reiter, Jana Striefler, Marco Blessmann, Thilo Hackert, Anna Duprée

**Affiliations:** 1 Department of General, Visceral, and Thoracic Surgery, University Medical Center Hamburg-Eppendorf, Hamburg, Germany; 2 Clinic and Polyclinic for Trauma Surgery and Orthopedics, University Medical Center Hamburg-Eppendorf, Hamburg, Germany; 3 2nd Medical Clinic and Polyclinic (Oncology, Hematology, Department of Pulmonology), University Medical Center Hamburg-Eppendorf, Hamburg, Germany; 4 Department of Plastic, Reconstructive, and Aesthetic Surgery, University Medical Center Hamburg-Eppendorf, Hamburg, Germany

**Keywords:** Vacuum assisted wound closure, Soft tissue sarcoma, Sarcoma surgery, Wound complication, Postoperative complications, Secondary wound closure

## Abstract

**Purpose:**

In sarcomas, surgery is an essential component of therapy. Depending on their location, sarcomas can reach a considerable size, which increases the risk of wound complications (WC) after resections. This often results in longer hospital stays and thus delays further oncological treatment. Therefore, reducing WCs is indispensable for improving treatment. The VAC (Vacuum-Assisted Closure) therapy has been shown to have a positive effect on wound healing, but there are limited studies for its use after sarcoma resections. The aim of this study was to analyze the outcomes of various wound closure techniques. This was intended to identify optimal wound care to prevent WCs and to determine risk factors for complications.

**Methods:**

This study is a single-center study that included all patients who underwent surgery for sarcomas of the body surface and extremities. A retrospective data analysis was conducted for the years 2021–2023. The primary endpoint was the development of wound complications. Here, primary wound closure was compared with secondary closure after negative wound pressure therapy (NWPT). Secondary endpoints included the impact of drains, subtype, location, and comorbidities.

**Results:**

A total of 211 patients were examined. The most common histological subtype was liposarcoma (32,88%), followed by undifferentiated sarcoma (19,18%). Wound complications occurred in 30,19% of all cases. The analysis showed that 40,4% of patients with primary wound closure developed a complication, while only 20% of patients with NWPT did. A significant risk factor for the development of a WC was a tumor diameter > 10 cm, which led to a 20,13% higher risk of infection compared to smaller tumors. 68.3% of wound complications occurred in the lower extremity. Additionally, neoadjuvant therapy, longer operation time and immunosuppression were detected as risk factors for a higher complication rate.

**Conclusions:**

This study highlights the significantly increased risk of WCs in large sarcomas of the lower extremities. VAC therapy showed a significant improvement in wound closure especially in high-risk cases. Based on the results, the use of NWPT can be essential for wound management in sarcomas and improve convalescence and oncological treatment.

## Introduction

Sarcomas are malignant diseases, which are rare but highly complex and heterogenous. They originate from the mesenchymal tissue and can appear anywhere on the body [1–3].The treatment of sarcomas requires close interdisciplinary cooperation. In most cases the therapy consists of surgery, followed by radiation or systemic therapy. Depending on their localization soft tissue sarcomas can reach significant sizes, making resections challenging and often resulting in large tissue defects. Consequently, wound complications (WCs) such as infections, seromas, lymphatic fistulas or wound dehiscence are common after sarcoma surgery. Studies report an overall wound complications rate of 20–42%, with rates influenced by factors such as patient age, tumor size and site, and preoperative treatment or comorbidities [4–7]. Notably, neoadjuvant radiation therapy contributes to a high wound complication rate of approximately 30%. This often leads to caution in its use, despite its positive impact on oncological outcomes [5, 8, 9]. 

Wound complications prolong hospitalization and negatively affect the patient’s overall condition. Therefore, subsequent treatments such as radiation or chemotherapy are often delayed in those cases. This can increase morbidity and adversely impact oncological outcomes. Thus, reduction of wound complications is essential for improving the perioperative care of sarcoma patients.

Currently, primary wound closure is the standard procedure following sarcoma resections. However, the use of advanced wound therapies such as “vacuum-assisted wound therapy”, is on the rise. The vacuum assisted wound therapy, also called negative wound pressure therapy, applies continuous suction to the wound, promoting faster healing by increasing blood flow, enhancing fibroblast and collagen migration, and removing wound exudate [10–13]. Additionally the hermetically seal created by the vacuum therapy helps reduce infections by prevention bacterial contamination, as noted by Deva et al. [10]. Previously, concerns existed that VAWT might increase recurrence rates, but several studies, including those in a meta-analysis by Wang et al., found no difference in local recurrence rates when compared to conventional wound dressings [14].

The first reference to vacuum assisted wound closure in soft tissue tumors was found in 2007. Siegel et al. evaluated the effectiveness of negative wound pressure therapy (NWPT) for radiation-associated wound complications in 22 patients, finding a significant reduction in hospital stay, overall treatment duration, and the need for tissue transposition for wound closure [13]. Following that, Bedi et al. studied the use of vacuum assisted wound dressing in soft tissue sarcoma resections following radiation therapy, reporting a reduction in wound infections from 49 to 9% in cases with preoperative radiation [5].

In 2023, Gusho et al. conducted a meta-analysis of four studies on NWPT for soft tissue sarcomas [15]. Notably, only one of these studies examined NWPT in place of wound closure; the remaining studies focused on using vacuum dressings on closed wounds [16–19]. The meta-analysis by Gusho et al. found an overall wound complication rate of 39.7% with conventional wound closure and dressing, which decreased to as low as 7.6% with NWPT [15]. 

Despite the promising findings, few studies specifically investigate the use of vacuum therapy within the resection cavity. In the last 20 years, only two studies have explored NWPT following musculoskeletal tumor resections, each with small sample sizes—23 patients in the study by Bickels et al. and 32 patients in the study by Sakellariou et al. [11]. – [20]. Additionally, these studies did not report on comprehensive NWPT data, including the correlation between wound complications, tumor characteristics like localization and size or patient demographics [11]. – [20].

Therefore, the objectives of this study are to identify risk factors for wound complications after soft tissue sarcoma resections and to assess the effectiveness of vacuum-assisted wound closure after sarcoma resections in a larger patient population. The goal is to provide recommendations for wound closure strategies that minimize complications.

## Methods

### Patients and study design

The study was conducted at the University Medical Center Hamburg-Eppendorf, Hamburg, a specialized sarcoma center, between January 2021 to December 2023. All patients over the age of 18 who underwent surgery at the center during this period were eligible for enrollment. Included in the study were patients who had resections of soft tissue tumors located in the upper and lower extremities as well as the trunk. Exclusion criteria included patients with intra-abdominal or intrathoracic sarcomas or those requiring soft tissue coverage. Based on these criteria, 211 patients were included in the study. The study was conducted in accordance with the Declaration of Helsinki and received approval from the local ethics committee in Hamburg. All participants provided written informed consent prior to enrollment.

### Clinical procedure

All patients were reviewed by a multidisciplinary tumor board and treatment recommendations were then presented to them. Surgical treatments were performed by specialized sarcoma surgeons according to oncological standards, with the primary aim of achieving negative surgical margins. All patients received preoperative antibiotics. Reconstructive plastic surgeons participated in cases requiring free flap reconstructions. Primary wound closure generally included layered closure and the use of drains when considered necessary by the surgeon. Depending on the tumor location, the wound was closed with simple interrupted sutures, staples, or continuous sutures. Vacuum-assisted closure (VAC) was mainly considered in cases with a high risk of external contamination, such as deep wounds, wounds under increased closure tension, or areas exposed to significant shearing forces. In some cases, VAC Therapy was also induced to cover the time until margins were examined by pathology. As this study was conducted retrospectively, there was no explicit protocol guiding the decision between NWPT (negative wound pressure therapy) and Primary closure, so the decision depended mainly on the surgeon performing the resection. The VAC sponge and dressings were applied under sterile conditions, with continuous suction set between − 75 and − 125 mmHg. −25mmHg was used when blood vessels were located in the resection area. The VAC dressing aimed to seal the wound, prevent external contamination, control fluid accumulation, and promote wound healing. Postoperatively, both deep and superficial drains were removed at the surgeon’s discretion. Patients typically retained NWPT for 5 to 7 days, after which a second surgery was conducted to inspect and close the wound with interrupted sutures if sufficient granulation was present. Sufficient granulation was determined by a clean, granular and soft tissue without necrosis or any signs of infections (bacterial coating). When the granulation process was disturbed or not satisfactory another period of NWPT was induced. This decision also depended on the surgeon performing the second surgery. Drains also were used at the surgeon’s discretion (e.g. in deep wounds, risk locations or preoperatively infected areas), and further vacuum treatment was applied if wound conditions required it, with dressings changed intraoperatively until satisfactory wound healing was achieved. Surgeons decided individually on the drainage duration. The main criteria for the removal were decreasing secretion and clear liquid.

### Clinical data

Data were collected from medical records and included patient demographics, tumor characteristics, treatment details, medical history, and closure techniques. All Data were managed using REDCap (Research Electronic Data Capture) tools hosted at the University Medical Center Hamburg-Eppendorf. The primary outcome measure was wound complication, defined as the occurrence of at least one of the following: infection, seroma, fistula, or wound dehiscence. Secondary outcomes included drainage output, duration of drainage treatment, location of wound infection, and other risk factors such as tumor size and comorbidities.

### Statistical analysis

All statistical analyses were performed using IBM SPSS Statistics for Windows, Version 19.0 (IBM Corp., Armonk, NY, USA), and graphs were generated using GraphPad Prism, Version 10 (GraphPad Software Corp., Boston, MA, USA). Absolute and relative frequencies were calculated for qualitative data, while quantitative results were represented by their median and standard deviation. The Shapiro-Wilk test was used to assess normal distribution. For wound outcomes, a dichotomous variable, the Phi coefficient was employed to assess the association between two binary variables. Comparisons between two groups were made using the Chi-squared test or Fisher’s exact test, as appropriate, and the Kruskal-Wallis test was used for comparisons involving more than two groups on a quantitative variable. Logistic regression analysis was conducted to evaluate the association between binary outcomes and quantitative explanatory variables (e.g., drainage volume). A p-value of less than 0.05 was considered statistically significant.

## Results

### Demographics

This study, conducted at the University Medical Center Hamburg-Eppendorf Sarcoma Center, evaluated data from 478 patients who underwent sarcoma resections. After applying exclusion criteria, 211 patients were included in the analysis. Table 1 presents the demographic and clinical characteristics of the study cohort. Among these patients, 124 (58.8%) were men, and 87 (41.2%) were women, with a mean age of 60.4 years (± 18.4).

Of the tumors, 146 were malignant, while 65 were benign, including myxomas and lipomas. The most common histologic subtype of malignant tumors was liposarcoma, observed in 48 cases (32.88%), followed by undifferentiated sarcoma (19.18%) and myxofibrosarcoma (17.81%). Most tumors were primary (82.94%), but also recurrent tumors (*n* = 29) and metastases (*n* = 14) were found in the cohort. The mean tumor size was 11.5 ± 6.96 cm. Tumors with a maximum diameter less than 10 cm accounted for 65 cases, while 81 tumors were larger than 10 cm. The majority of tumors were located in the lower extremities (*n* = 116), followed by the trunk (*n* = 56) and the upper extremities (*n* = 35).

### Occurrence and predisposing factors of wound complications

Among the 211 patients, 30.19% experienced wound complications, such as seromas, infections, dehiscences, or lymph fistulas. The most common complication was wound infection, which affected 33 patients; 32 of these patients also developed seromas. Based on the Clavien-Dindo Classification, 17.1% of patients experienced minor wound complications (Grade 1–2), while 15.8% had major complications (Grade 3–4).

Several potential risk factors for wound complications (WCs) were evaluated (Table 1). No significant correlation was found between WC rates and either patient sex or age. The evaluation of the use of noxious agents like smoking and alcohol also showed no significant correlation to wound complications. However, the group of patients without complications contained 24% smokers while the group of patients with complications had a higher percentage of smokers with 30,23%. When comparing other clinicopathological characteristics between patients with and without complications no significant differences in BMI, ECOG Status, diabetes or preoperative laboratory values (e.g., leukocyte count and CRP). However, univariate analysis showed a significant association between pre-existing immunosuppression (due to medication or immunosuppressive conditions) and WC development (*p* < 0.05).

Primary tumors led to complications in 46 patients (26.29% of primary tumors), while recurrent and metastasised cases had complication rates of 34.48% and 35.71%, respectively. Tumors larger than 10 cm were associated with a 20.13% increased risk of infection compared to smaller tumors, resulting in a significantly higher risk of WCs at T3-4 stages (*p* = 0.003).

**Table 1:**
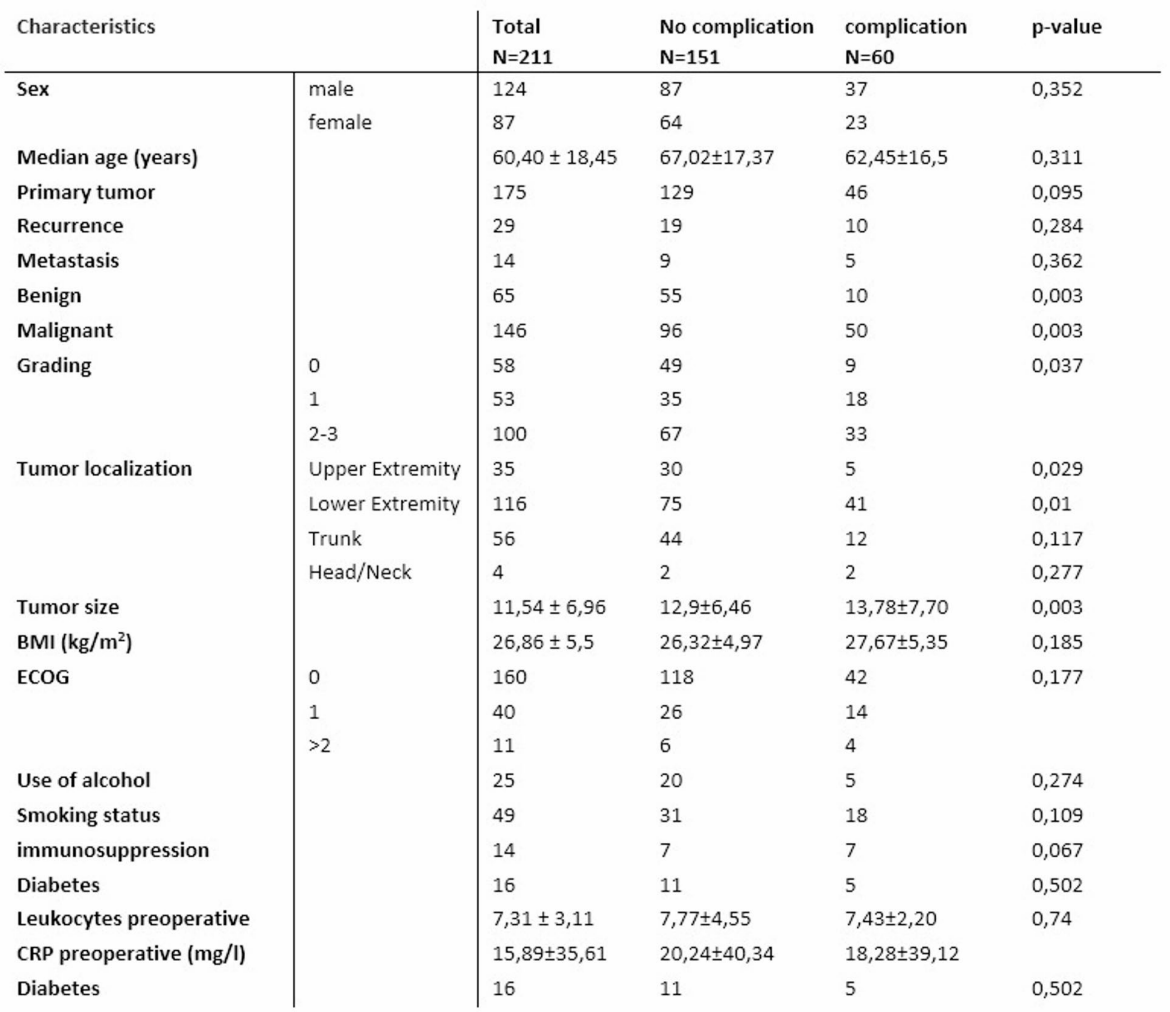
Characteristics of the patient cohort (all tumor) and two subgroups (patients with and without complications)

**Fig. 1:**
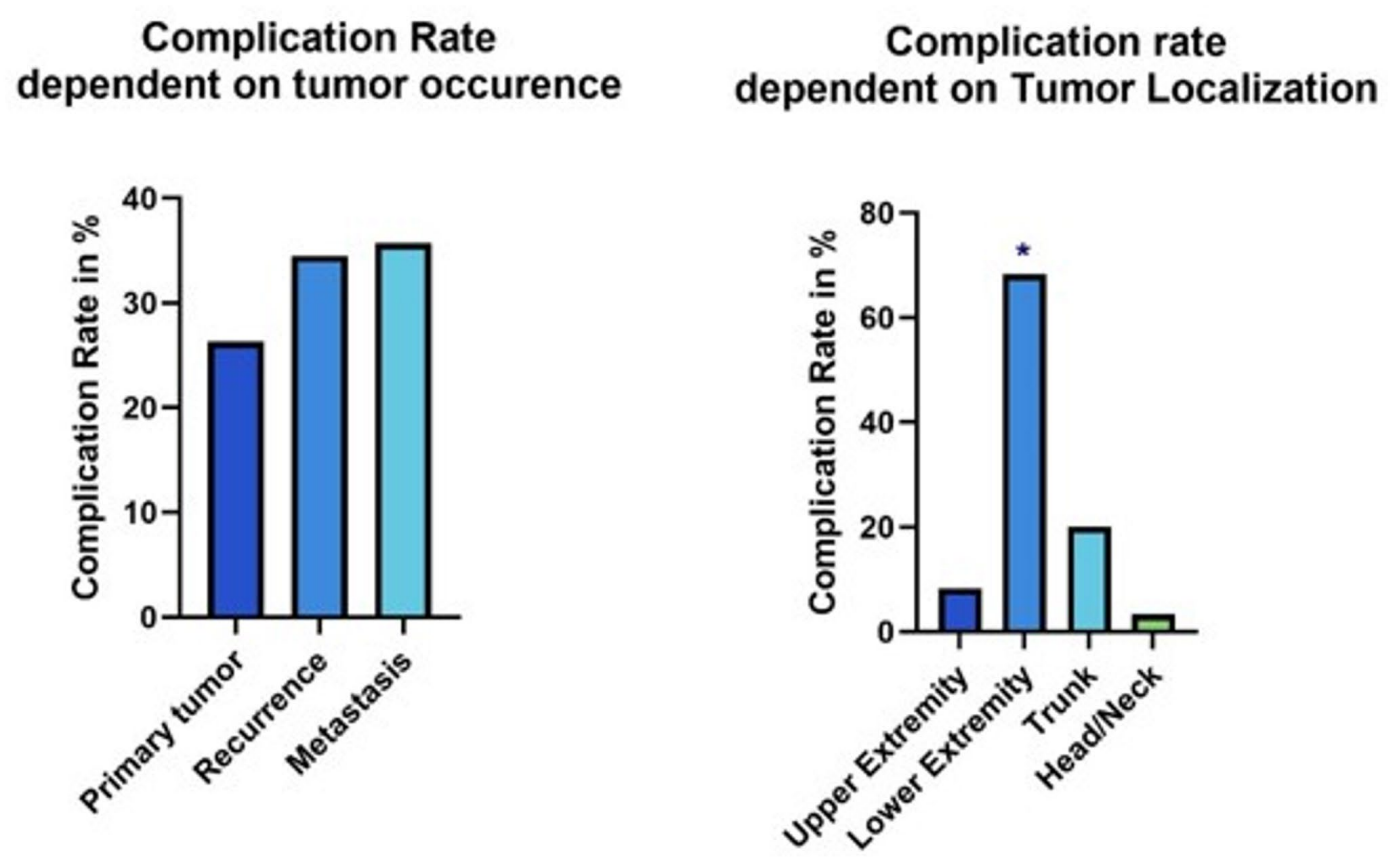
Risk factors for wound complications

**Fig. 2:**
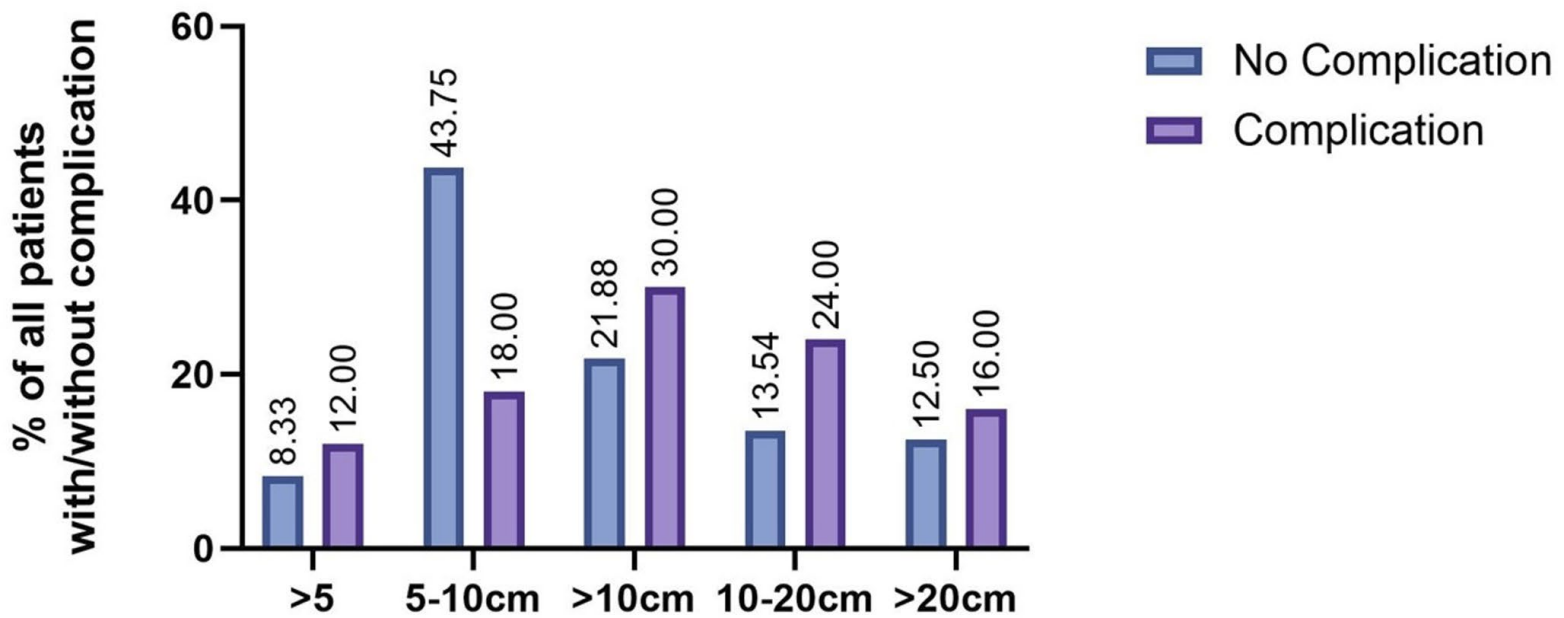
Tumor Size as a Risk factor for wound complications: Increased Risk of WCs in Tumors > 10 cm (30% of all patients), 10–20 cm (24% of all patients) and > 20 cm (16% of all patients)

Additionally, 68.3% of WCs occurred in lower extremity tumors, compared to 8.3% in the upper extremities and 20.0% in the trunk. Thus, tumors of the lower extremities had a significantly higher risk of complications (*p* = 0.01). *(See* Figs. 1 and 2)

To evaluate the risks associated with different tumor sizes in the lower extremity, identified as a high-risk area, the cohort was divided into four subgroups: 1: Maximum tumor diameter < 5 cm; 2: Maximum tumor diameter 5–10 cm; 3: Maximum tumor diameter 10–15 cm; 4: Maximum tumor diameter > 15 cm. Among these subgroups, the complication rate increased in correlation with tumor size (29.17% in tumors from 5 to 10 cm and 42.2% in tumors > 15 cm). However, there was no significant difference in the total number of complications across the groups.

When analyzing tumor grading, no significant difference in WC rates was found between higher-grade tumors (G2-3) and lower-grade tumors (G1) for both the complication and non-complication groups. Overall, dedifferentiated liposarcoma had the highest WC rate (23.68%), followed by undifferentiated sarcoma (21.05%), with these subtypes showing a WC risk of 50–56%.

In the assessment of risk factors in treatment, a significant difference regarding the operation time was detected. The operations of patients without complications lasted a median of 74,63 ± 58,35 min while those with following complications lasted 113,47 ± 74,57 min (*p* = 0,001). Other variables, such as previous surgeries or vascular involvement during surgery, were not associated with increased WC rates.

Perioperative treatment was influential as well. While chemotherapy (neoadjuvant or adjuvant) did not significantly affect WC rates, local radiation had a notable impact. Of patients receiving neoadjuvant radiation, 61.54% developed WCs (*p* = 0.011), compared to a 37.21% WC rate in those receiving adjuvant radiation.

## Comparison of different wound closures

**Fig. 3:**
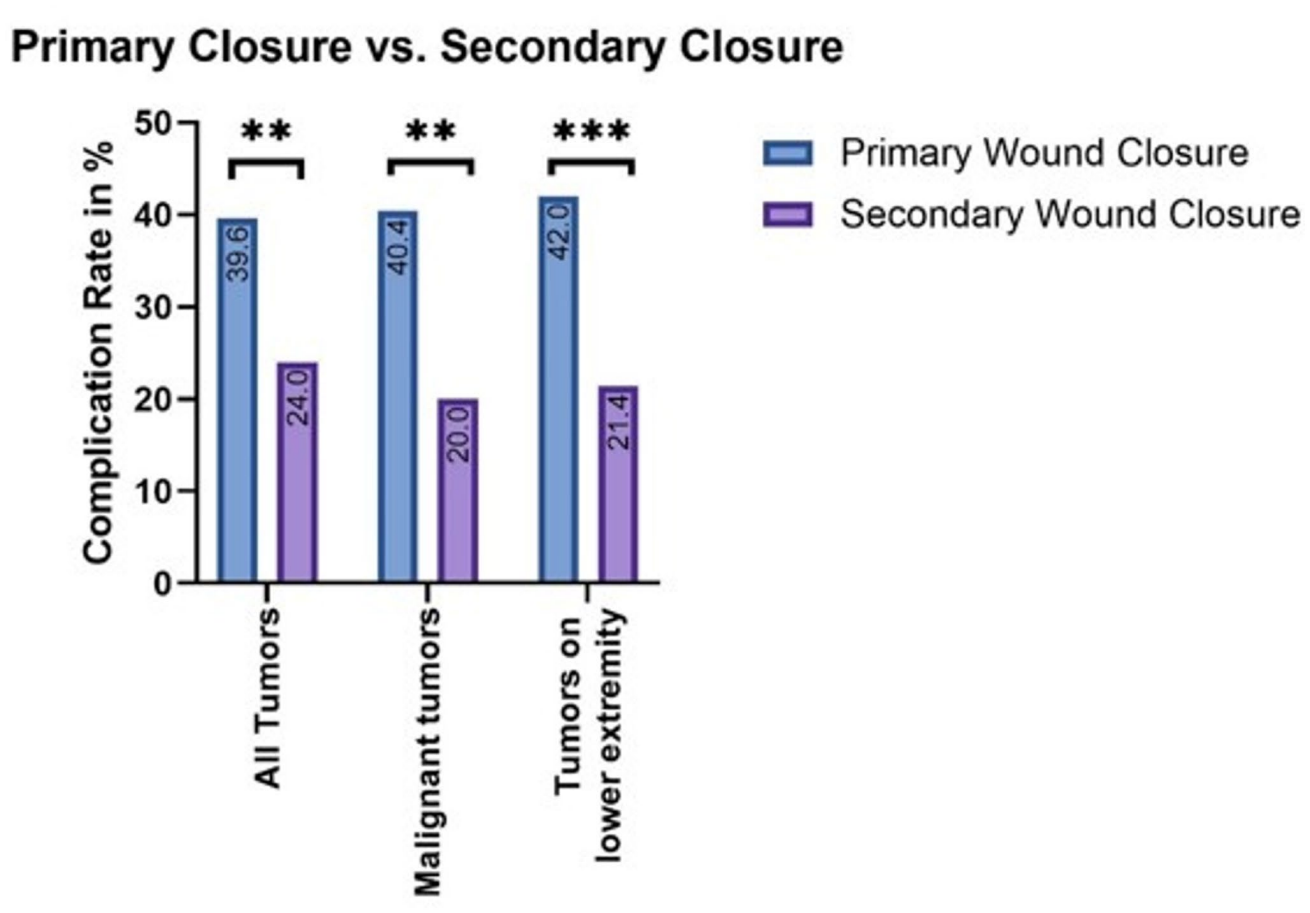
Wound complication rate after primary and secondary wound closure in all tumors (significant decrease of WCs from 39,6–24%); only malignant tumors (Decrease from 40,4–20%); tumors on the lower extremity (Decrease from 42% to 21,4%)

Primary wound closure was associated with a 30.19% rate of wound infections, wound dehiscence, or seroma across all soft tissue tumors. Malignant soft tissue sarcomas had a significantly higher complication rate of 40.4% (*p* = 0.0122). By contrast, patients who received vacuum-assisted wound closure (VAC) had a lower WC rate of 20%, a reduction of 20.4% compared to standard closure. By using the NWPT the rate was reduced by 20.4%. The Odds Ratio for a WC without using vacuum therapy after resections of malignant soft tissue sarcomas was significant with 2.71. In the high-risk location of the lower extremity the complication rate was reduced from 42.0 to 21.4% with a significant Odds Ratio of 2.66. *(See* Fig. 3*)*

**Fig. 4:**
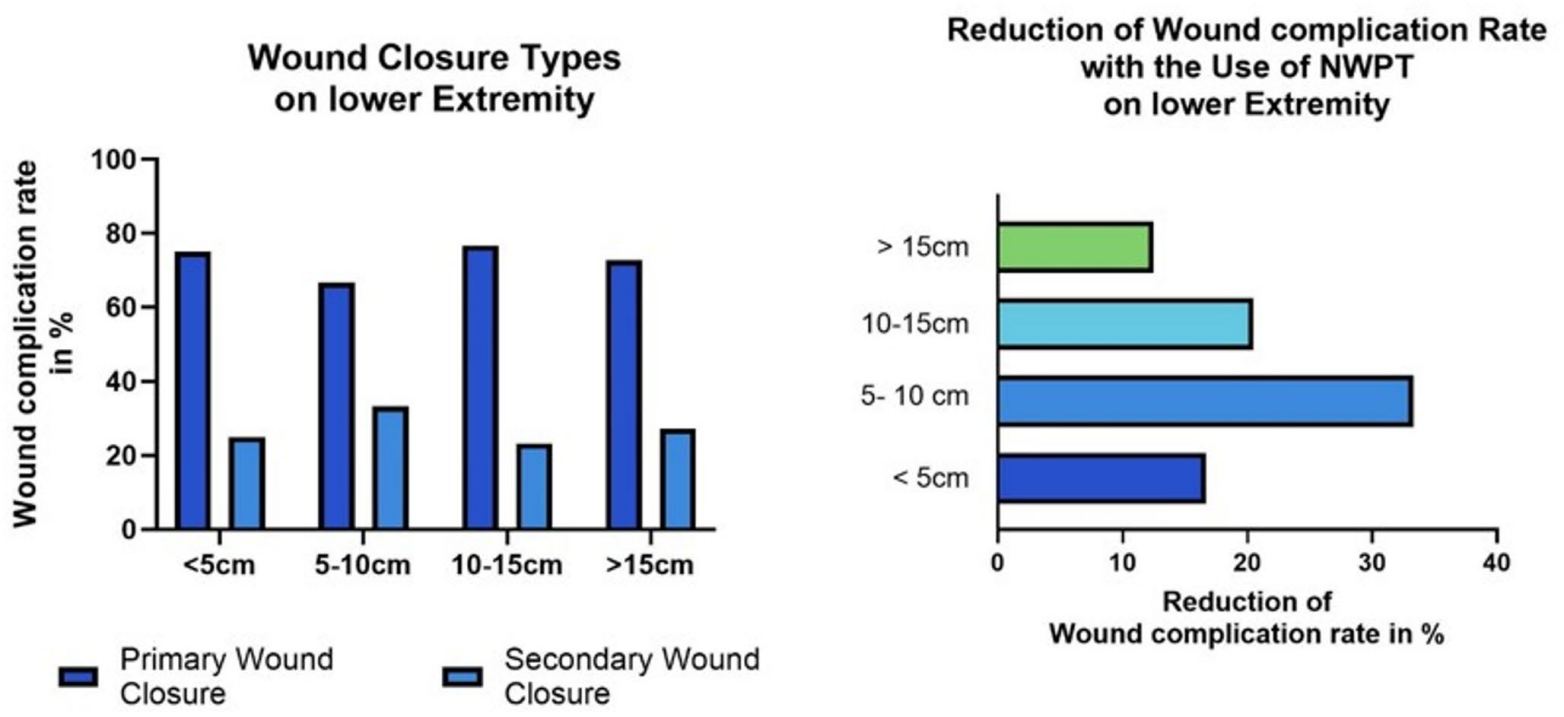
Wound complication rate after primary and secondary wound closure in subgroup analysis: **a**)wound complication rate in tumors on the lower extremity- reduction of wound complication rate in all size subgroups. **b**) Reduction of wound complication rate on tumors of the lower extremity shows the strongest reduction in tumors from 5-10cm, followed by tumors from 10-15cm

Once again, the different subgroups were evaluated. The use of vacuum therapy varied among the subgroups. For example, while only 18.2% of the tumors smaller than 5 cm were treated with NPWT, after the resection of tumors from 5 to 10 cm, vacuum therapy was used in 33.4% of cases. All subgroups showed a significant decrease in wound complications following the use of vacuum therapy. In the first group (tumors < 5 cm), the complication rate was reduced from 66.7 to 0.5% (OR: 1,33). In the second group, the rate was reduced from 37.5 to 12.5% (OR: 3), and in the third group, it went from 34.78 to 14.29% (OR: 2,43). The largest tumors in the fourth group showed a smaller reduction in wound complication rates, with vacuum therapy decreasing the rate from 45.83 to 33.34% (OR: 1,375). (See Fig. 4) This shows that the risk to develop a wound complication was more than doubled in group 2 and 3.

**Fig. 5:**
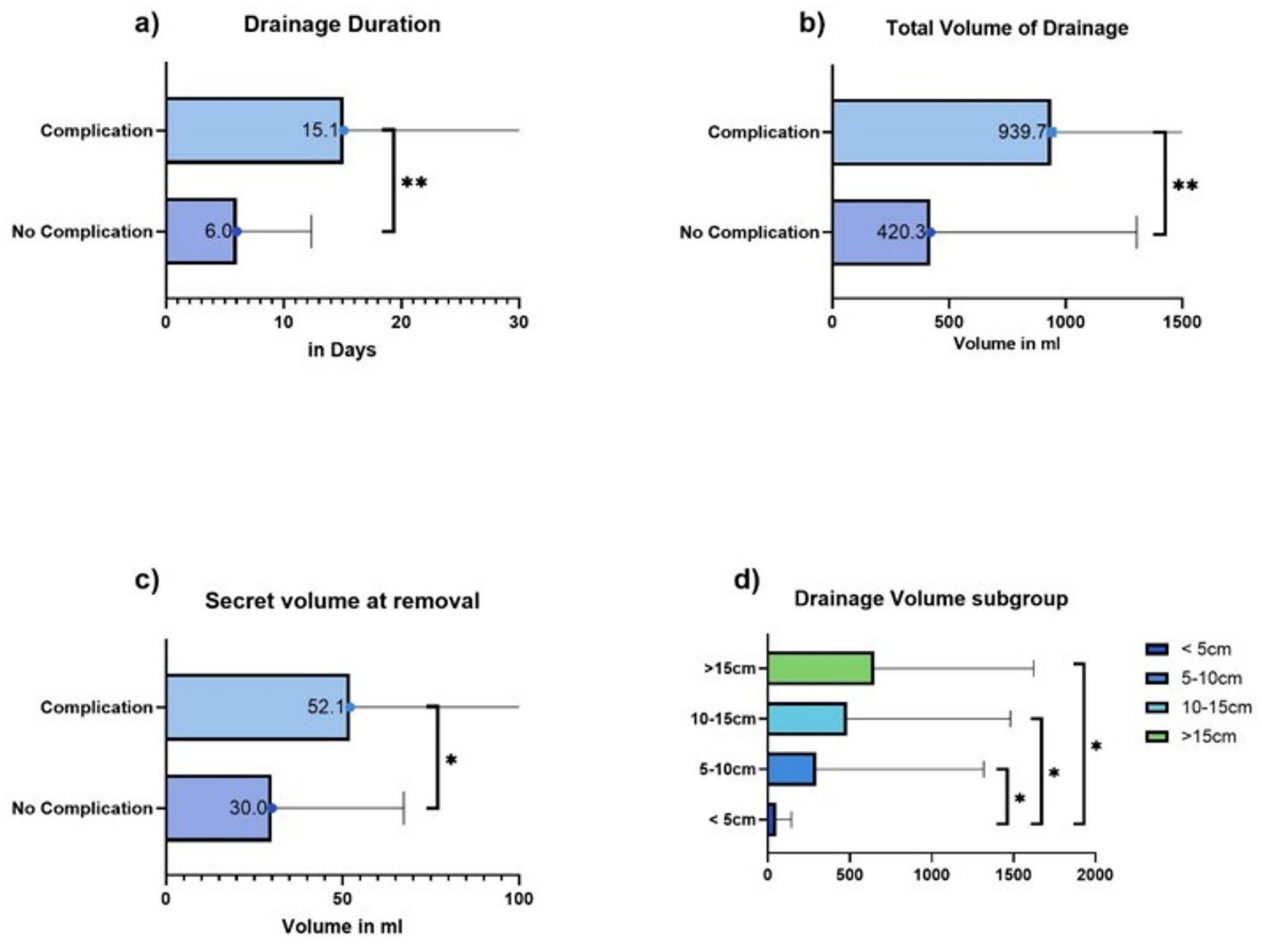
The use of Drainages after sarcoma resections in patients with and without complications: **a**) duration of drainage use in days, significant reduction of drainage duration in patients without complications, **b**) total volume of drainage of drainage was significantly higher in patients with complications, **c**) drainage volume at day of removal was significantly higher in patients with a complication, **d**) Total drainage volume after resection of different sized tumors, significantly increased drainage secretion after resection of bigger tumors

Drainages were used in 63.7% of malignant sarcoma cases (93 patients) and were typically removed after 6.82 ± 15.75 days, with a median drainage output of 25.68 ± 41.53 ml. Total drainage volume varied widely, with a median of 424.21 ± 922.57 ml. Under omission of the patients who received plastic surgery, the secret volume of the drainages showed a correlation with wound complications. Patients with drainage outputs over 50 ml at the time of removal had a complication rate of 57.1%, compared to 27.4% for those removed under 50 ml (*p* < 0.01). This also included the patients who received a secondary wound closure beforehand. Total drainage output volume was also significantly correlated with WC rates; patients without complications had a median output of 359.81 ± 771.78 ml, while those with complications had 961.82 ± 1197.55 ml (*p* = 0.001). Matching these data the length of the drainage duration was significantly higher in patients with a complication (*p* = 0,003). *(See* Figs. 5a-c*)*

In the different size subgroups, the drainage volume also differed immensely. After resection of the smallest tumors the total drainage volume was 50 ± 92,58 ml. All the other tumor resections resulted in a significantly higher total drainage volume which also increased with the size of the tumors. (Group 2: 293,71 ± 1024,4 ml; Group 3: 483,67 ± 996,64 ml; Group 4: 650,91 ± 971,96 ml). *(See* Fig. 5d*)*

**Fig. 6:**
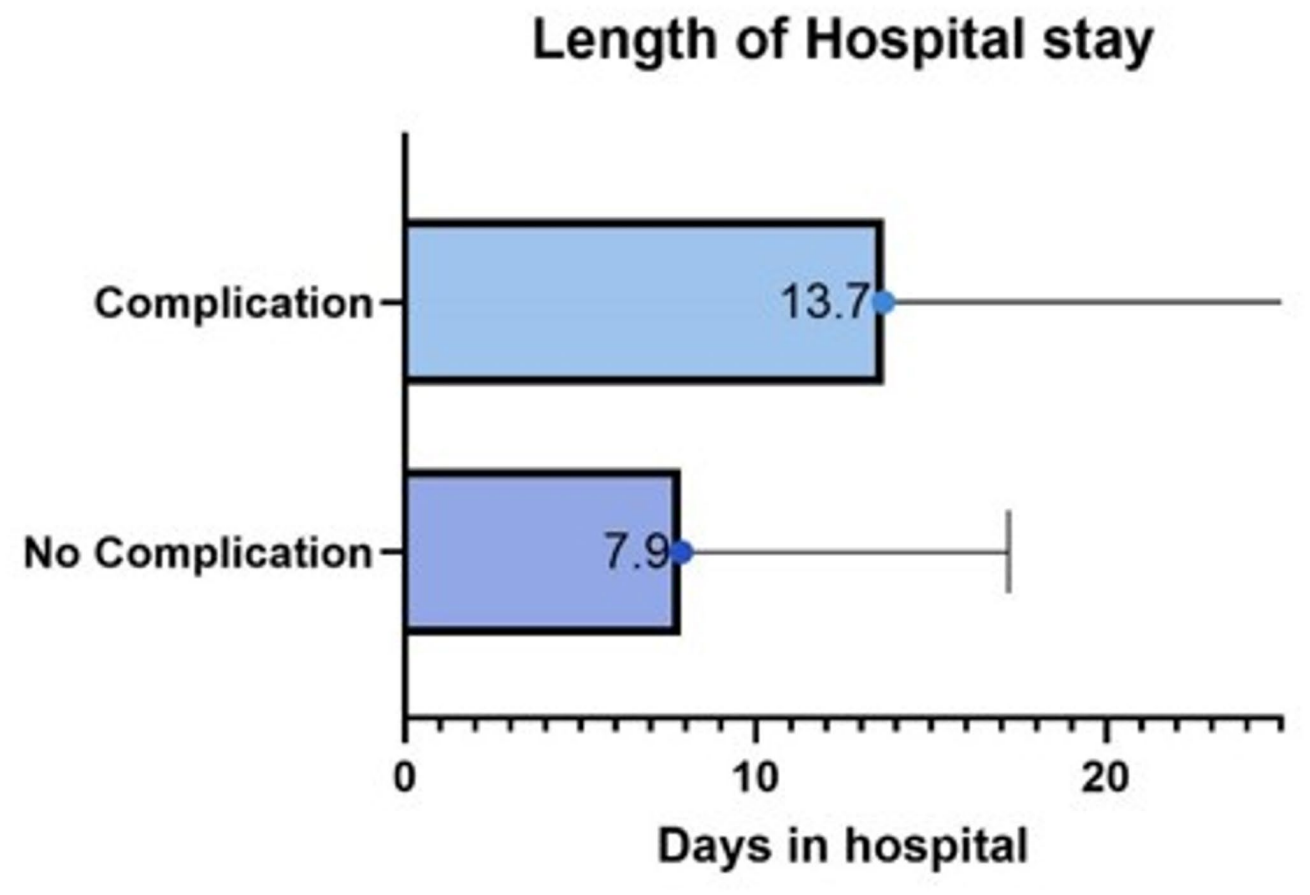
Hospitalization of patients with and without wound complications: Patients with complications had an overall longer hospital stay (13,7 days) than patients without wound complications (7,9 days)

The mean hospital stay was 14.38 ± 17.03 days. Excluding patients who underwent plastic reconstruction, the median stay was 11.33 ± 12.36 days. Extended hospitalization was significantly correlated with WCs; patients without complications had a mean stay of 8.59 ± 9.13 days, whereas those with complications averaged 14.79 ± 16.04 days (*p* < 0.05). Patients receiving NWPT had a longer mean hospital stay (20.58 ± 11.76 days) compared to those without (9.67 ± 11.8 days, *p* = 0.004), with the vacuum therapy typically applied for 11.32 ± 7.13 days. *(See* Fig. 6*)*

Regarding oncological outcomes, R0 resection status was achieved in 90.6% of patients with primary wound closure but only 66% in the VAC group after the initial surgery. Secondary wound closure allowed for re-resection to achieve tumor-free margins when necessary and was used for that purpose in the described cohort. After wound healing, 8.3% of patients with primary closure received adjuvant chemotherapy, while 19.8% received adjuvant radiation. In the VAC group, 10% received chemotherapy, and 48% received postoperative radiation.

## Discussion

The primary objective of this study was to compare the outcomes of soft tissue sarcoma surgery with primary and secondary wound closures in terms of complication rates, length of hospital stays, and oncologic outcomes. Moreover, this study was conducted to identify risk factors for the occurrence of wound complications. The findings suggest that secondary wound closure with intermediate NWPT offers significant advantages in avoiding wound complications. However, it also has limitations and should therefore be used mainly for high-risk patients.

### Risk factors for wound complications

In comparing all complications, one main risk factor for wound complications was malignancy. While the rate of wound complications in all patients with soft tissue tumors was 28,4, the occurrence of a malignant tumor, a soft tissue sarcoma, led to a significant increase to a WC rate of 34,24%. This matches the WC rate in previous studies [21]– [22]. One of the main risk factors mentioned in literature is the tumor volume [21, 22]. In our study there was a significant increase in WC rates from 23,08% in tumors < 10 cm to 43,21% in tumors > 10 cm. This increase in wound complications can be due to bigger tumor resection cavities, which are filled with wound liquids and induce infections or seromas. This was also shown by the significantly higher amount of drainage volume in the larger tumors as seen in the different subgroups in this study.

Another main risk factor found in this study was the localization of the tumor on the lower extremity and the groin. A total of 74.4% of complications developed in this area, and even in small tumors < 5 cm, 76.8% of the complications occurred on the lower extremity. In the lower extremity there was a significantly higher complication rate in the bigger tumors > 10 cm (65,71%) versus the smaller tumors < 10 cm with a complication rate of 34,29%. The higher overall WC rate on the lower extremity might also be caused by difficulties in maintaining optimal wound hygiene in this area of the body. Other studies focus on other predictors of wound complications, such as tumor presentation (recurrent/metastasized vs. primary tumor) [23]. However, this aspect was not significantly relevant in our study, with a 26,29% complication rate in the primary tumors and 35,71% in the metastases.

Another important aspect in the development of wound complications is the oncological treatment. Several studies discuss wound management after local radiation of STS [5, 8, 9]. Radiation has been proven to significantly increase the risk for wound complications. This study validates this fact and demonstrates a significantly high complication rate of 61,54% in patients who received neoadjuvant radiation. This was apparent in tumors of several locations.

Patients’ characteristics such as gender and age did not play a role in the development of WC, as confirmed both by the literature and by our study [22–27]. Most previous studies evaluated the influence of comorbid conditions like diabetes, obesity or smoking. Some studies found a significant increase in complications among patients with these conditions [24, 27]; however, most studies did not show a significant correlation. In this study, there was also no significant increased risk associated with these factors. The only comorbidity resulting in an increased WC rate was immunosuppression due to medication or disease, a factor proven to be highly relevant in wound healing, although this was not assessed in other studies regarding WC in STS [26].

Defining the main important risk factors for wound complications is essential in establishing an optimized wound treatment for each tumor. Certainly, the localization as well as the tumor size can be considered main aspects. Accordingly, high-risk STS for WC are those on the lower extremity with a diameter of > 10 cm. Additionally, neoadjuvant radiation significantly increases the risk for WC.

### Benefits and disadvantages of the vacuum assisted wound closure

This study demonstrated a significant decrease in wound complications after using NWPT. Following primary wound closure, 30,19% of all patients developed a wound complication. With malignancy as a risk factor for a higher WC rate there was a significant increase to a rate of 40,4% in the STS cases. After applying NWPT and performing secondary wound closure, only 20% of the STS patients developed a wound complication, marking a significant decrease.

A few previous studies examine the use of vacuum therapy in STS. The mentioned meta-analysis by Gusho et al. discussed 4 studies, from which none covers NWPT in the tumor cavity but examined the use of vacuum dressings on closed incisions after sarcoma resections. Shields et al. found that NPWT on closed incisions appears to result in lower surgical site infection rates than conventional dressings, though due to a low number of patients, these results were not statistically significant [16]. Dadras et al. also investigated the use of close incision negative wound drainages in benign and malignant soft tissue tumors, finding a reduction in wound infections to only 3% (control: 13%). This also correlated with a reduction in drainage volume und length of hospital stay. Nevertheless, seroma formation did not occur less in these patients, which may be due to the depth of wound cavities that could not be reached by closed incision vacuum therapy [17]. Other studies evaluating closed-incision vacuum therapy have reached similar conclusions [18]– [19]. However, as shown by Dadras et al. especially the seroma formation could not be reduced by closed-incision vacuum therapy even though a drain was used if needed. This was true especially for tumors with a larger depth. As seen in our cohort, 53% of all complications without the use of NWPT were also seromas and the overall complication rate was increased in correlation to the tumor size and the wound cavity size.

In this study the NWPT was used in the tumor cavity. While 17,6% of the patients without vacuum therapy developed seroma, only 7,7% developed one after a NWPT. This shows a significant impact of vacuum therapy in the tumor cavity (*p* = 0,0122). One can conclude that especially patients with large tumors and resulting large tumor cavities profit from NWPT. Due to these findings, a prospective randomized trial has recently been initiated to examine closed-incision vacuum therapy [27].

Overall, the literature shows studies, in which the complication rate was reduced to the same amount of WC only by performing the incisional VAC Therapy [15]. However, all the studies had smaller patient populations or concentrated on specific risk factor populations like patients after radiation.

Another risk factor for the development of wound complications (WC) was a tumor diameter greater than 10 cm. As demonstrated in this study, NPWT can also reduce WC in such cases. With an overall WC rate of 43.1% after primary wound closure and 27.8% after secondary wound closure, NPWT has been shown to significantly improve wound healing in tumors smaller than 10 cm. When combining the risk factor of localization in the lower extremity with larger tumors, an even greater reduction in wound complication rates was observed. In the group of 5–10 cm tumors, the complication rate decreased from 66.67 to 33.34% (−33.34%), and in the group of 10–15 cm tumors, the rate was reduced from 34.78 to 14.28% (−20.5%) with the use of NPWT. In tumors < 5 cm the rate was only reduced by 16,67%. However, these subgroups were too small to demonstrate a statistically significant effect of vacuum therapy.

Moreover, most WC occurred on the lower extremity, a trend confirmed by previous studies [4–6]. In this study, vacuum therapy reduced WC from 42% to 21,4% also on the lower extremity, which was highly significant (*p* = 0,0038).

As already noted, neoadjuvant radiation is another important risk factor for WC. Bedi et al. demonstrated that NWPT on the closed incision is associated with a lower risk of wound complications in patients after radiation5.

In this study the WC rate of patients with neoadjuvant radiation was reduced from 62,5 to 50% by using vacuum therapy. Although this reduction was not statistically significant, this suggests an improvement in wound healing even in irradiated regions also using NWPT in the tumor cavity.

One main concern with NWPT in oncological patients is the potential of tumor cell distribution by the procedure. However, in randomized and retrospective studies on NWPT, no data were reported on the development of local recurrence, metastasis, or disease progression [28, 29]. Fourman et al. used temporary vacuum therapy in 62 patients in the resection bed of soft tissue sarcomas until final margins were achieved, reporting a modest local recurrence rate of 8.1% after 2 years, similar to recurrence rates following conventional wound closure [26]. The practice of temporized vacuum therapy while awaiting final pathology margins has also been examined in other studies, which similarly demonstrate the oncological safety of this method [30]– [31].

Because NWPT was applied in the tumor cavity, the patients needed a second surgery to perform the secondary wound closure. This second surgery could present a risk factor for higher morbidity and mortality. However, several studies show that anesthesia and elective surgeries are safe when thorough risk assessment is performed [32, 33]. Increased complication rates are associated with higher patient morbidity (ASA III-IV). In this study, mostly patients with an ASA I-II status were examined. In the case of a multimorbid patient, the risk of a second surgery must be evaluated and weight up against the risk of a wound complication.

Another important goal in optimal wound management is faster convalescence and earlier initiation of other oncological treatments. Shorter hospital stays are associated with an improved oncological outcome and lower morbidity and mortality [34, 35]. In this study NWPT resulted in a significant longer hospital stay. In this cohort this could be due to the use of vacuum therapy until negative margins were achieved and its use in high-risk patients. Nevertheless, there was also a significant increase in hospitalization in patients with wound complications. In clinical practice, discharging patients with vacuum therapy can help reduce hospital stay duration. NWPT use in outpatients is approved and safe, especially benefiting young, mobile patients who can be discharged for a few days between surgeries [36]. In this context one also has to discuss the cost- effectiveness and resource utilization of NWPT. While the positive effect on wound healing can be proven in several studies, the argument of additional surgeries and more complex wound therapy can be debated. A large meta-analysis by Vikatmaa showed that NWPT costs less or at least similar to the control therapy in several studies [37]. However, the costs of secondary surgeries to perform the wound closure were not assessed. Since the second therapy stands in contrast to a longer hospitalization in cases of WC it is crucial to address this question in further studies.

All in all, this study demonstrated several advantages of NWPT. Vacuum therapy can reduce wound complications and improve the wound management, especially in high-risk patients.

### Limitations

As this study is a monocentric and retrospective examination, the results have certain limitations. The Data collection included the relevant risk factors and known manifestations of postoperative wound healing disorders. However, other factors, that were not assessed may also be important. Further prospective studies are needed to minimize bias, such as the more frequent use of NWPT in more challenging wounds or wounds which cannot be closed primarily. Additionally, the therapy with vacuum-assisted devices was performed with considerable variation in therapy duration, as well as the amount and frequency of dressing changes. This protocol variability adds a highly important bias to this study. Moreover, due to retrospective design, the selection of the population and the decision to perform the NWPT is influenced by the surgeons, which also supports the need for a prospective study design with standardized protocols. However, this study’s population is larger than those in other recent studies investigating wound complications after the resection of soft tissue tumors. Therefore, the study can be seen as a representation of a variety of patients with VAC Therapy, and the identified significant risk factors, as well as difference in wound management protocols, can be considered minor.

In addition to that, the study brings up the question of feasibility and cost-effectiveness of NWPT. While the NWPT leads to higher costs through additional surgeries and the need for devices, the shorter hospitalization and reduction of WC can also lead to a reduction of costs in the long run. These aspects should be addressed in further studies.

In summary, the retrospective study should be followed by a prospective randomized study to evaluate the wound closure types with minimized bias and focus on further aspects of the therapy.

## Conclusion

Our center’s analysis demonstrated that resections of larger sarcomas in the lower extremity are associated with a significantly increased risk of complications. However, the use of vacuum therapy and secondary wound closure reduced the wound complication rates by 20% on average. This retrospective study provided solid evidence of the benefits of NWPT, particularly for wounds on the lower extremity with a diameter of > 10 cm. High risk wounds following radiation treatment may also benefit from the use of NWPT. Additionally, the use of a drainage in larger wound cavities is essential and should not be removed if secretions exceed 50 ml. The decrease of WC rates can be compared to incisional VAC therapy, however, the NWPT in the tumor cavity might be superior to improve wound management after lager tumor resections. The study´s data confirms the significance of optimal wound management to prevent prolonged hospitalization and delays in further oncological therapy.

The retrospective design of this study limits its ability to make strong statements or guideline recommendations. However, this project lays the groundwork for further prospective, multicenter studies which are already planned by the University Medical Center Hamburg-Eppendorf in collaboration with the German Sarcoma Group. The ongoing SAVE Study explores the use of negative pressure wound therapy (NPWT) in tumor cavities following the resection of tumors larger than 5 cm in diameter located in the groin and lower extremities. The findings may help validate the anticipated benefits of vacuum-assisted wound closure in improving postoperative wound management.

## Data Availability

Data from medical records are not publicly available as they contain sensitive information. Register data may be available upon request to s.schewe@uke.de.

## Abbreviations

WC: Wound complications
STS: Soft tissue sarcoma
NWPT: Negative wound pressure therapy
DFSP: Dermatofibrosarcoma protuberans
MFS: Myxofibrosarcoma
